# Targeting Cancer With Bifunctional Peptides: Mechanism of Cell Entry and Inciting Cell Death

**DOI:** 10.1111/cas.70065

**Published:** 2025-03-26

**Authors:** Maria F. Setiawan, Oliver Rudan, Ingo G. H. Schmidt‐Wolf

**Affiliations:** ^1^ Department for Integrated Oncology University Hospital of Bonn Bonn Germany

**Keywords:** bifunctional peptide, cancer, clathrin‐mediated endocytosis, MLKL, NMIIA

## Abstract

Antimicrobial peptides have gained much attention in clinical research due to their extensive possibilities of application beyond antimicrobial use. The modification of antimicrobial peptides enables the peptides to target particular cancer cells, improving the specificity and efficiency of the treatment. In this study, TP2‐*D‐*Tox, a derivative of TP‐*D‐*Tox, demonstrated a superior anti‐tumor activity towards renal carcinoma, Caki‐2, and breast carcinoma, SK‐BR‐3. TP‐Tox was previously reported to inhibit tumor growth in a mouse model, increasing the overall survival. TP‐ and TP2‐*D‐*Tox were shown to penetrate the cells via clathrin‐mediated endocytosis, triggered by binding to the subunits of non‐muscle myosin IIa and S100A9. HSPB1 was observed to have a protective effect towards TP2‐*D‐*Tox against the immediate proteolytic inactivation. The intracellular presence of the peptides evoked mitochondrial permeability transition, generation of reactive oxygen species, and formation of MLKL oligomers in the plasma membrane. Our investigation revealed that TP‐ and TP2‐*D‐*Tox induced a similar but distinctly regulated cell death in Caki‐2 and SK‐BR‐3 cells. Both peptides established toxicity without activating any caspases, suggesting the possibility of TP‐ and TP2‐*D‐*Tox as a promising approach to bypass the caspase‐dependent apoptosis‐resistance issue impairing therapeutic responses of many cancer treatments.

## Graphical abstract

()‐D‐Tox peptide binding to the ligands triggers their internalization via clathrin‐mediated endocytosis. Intracellular presence of ()‐D‐Tox peptide induces a transient loss of mitochondrial membrane integrity and oligomerization of MLKL. Translocation of the oligomers to the plasma membrane causes a leakage of the cytosolic content and ultimately leads to cell death.

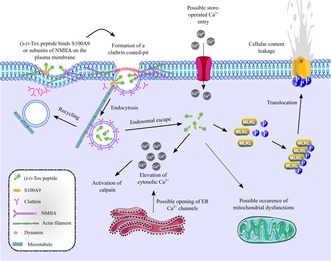

**Abbreviations**
8‐OHG: 8‐hydroxyguanosine
AMP: antimicrobial peptide
ATCC: American Type Culture Collection
CCCP: carbonyl cyanide m‐chlorophenyl hydrazone
CME: clathrin‐mediated endocytosis
CPZ: chlorpromazine
DsiRNA: dicer‐substrate short interfering RNA
ELC: essential light chain
G3BP1: ras GTPase activating protein‐binding protein 1
IC_50_
: half‐maximal inhibitory concentration
MLKL: mixed lineage kinase domain like pseudokinase
NMII: non‐muscle myosin II
RLC: regulatory light chain

## 1 Introduction

The discovery of antimicrobial peptide (AMP) expanded our horizon of therapeutic means against various insults and diseases. A sum of 5099 peptides have been registered in the AMP database, updated in January of 2025. AMPs are found in all six kingdoms of life and are diverse in structure, activity, and amino acid species. However, they share similarities in having short residues between 10 and 90 amino acids and a majority of AMPs being cationic [1, 2].

A 14 amino acid AMP, (KLAKLAK)2, was designed to possess the amphipathic α‐helical domain, which is commonly found in naturally occurring AMPs with biological activity. This peptide has a high polarity and is therefore hardly able to cross the plasma membrane. Due to its overall properties, the (KLAKLAK)2 peptide showed a high bacteriostatic activity and a considerably lower cytotoxicity towards human erythrocytes [3]. Nonetheless, introducing this peptide to human sarcoma and melanoma cell lines at 1% of their inhibitory concentrations (IC_50_) was described to induce mitochondrial swelling prior to cell rounding. Upon this finding, many studies attempted to couple (KLAKLAK)2 to a non‐toxic peptide with tumor‐homing properties. The targeting domain was included to guide the pro‐apoptotic peptide towards the targeted cells and facilitate their entry into the cells. The resulting bifunctional peptides were reported to have a lower IC_50_ for their particular targeted cells when compared to their corresponding monomers, to alter mitochondria, and to cause cell death. When introduced into tumor‐bearing mouse models, the peptides reduced the tumor volume and metastatic burden, promoting overall survival [4, 5, 6].

ErbB2 is a tyrosine kinase receptor without any known specific ligands. The unique structure of its extracellular domain encourages other ErbB receptors to bind to the open conformation of the outer domain. The resulting dimers strengthen the kinase‐mediated activation of the downstream signaling pathways and subsequently disrupt the control of the cell cycle‐promoting oncogenesis. Among the more aggressive tumor types, overexpression of ErbB2 has been associated with a lower prognosis and a shorter disease‐free interval. Therefore, targeted therapies against this protein are vastly investigated [7].

Numerous drugs targeting ErbB2 have been approved by the Food and Drug Administration and are widely used in clinical settings. Transtuzumab and Lapatinib are proven to hamper ErbB2 signaling and thus improve patient prognoses. Nevertheless, these antibody therapies do not always achieve suppression of malignancies. Transtuzumab has difficulties penetrating tumors of a particular size, while Lapatinib has a low bioavailability in certain human tissues [8, 9]. To overcome these issues, several studies designed short peptides capable of targeting ErbB2 and conjugated them to a drug that was intracellularly toxic. The peptide LTVSPWY was selected from a random peptide phage library due to its affinity to the ErbB2‐expressing cell line SK‐BR‐3. This phage peptide could successfully deliver an antisense oligonucleotide of ErbB2 into SK‐BR‐3 cells. Coupling this peptide to (KLAKLAK)2 allowed the new peptide conjugate TP‐Tox to acquire toxic activity inside the target cells. Intriguingly, the TP‐Tox peptide was shown to induce cell death in several solid tumor cell lines regardless of their ErbB2 expression and furthermore increased the survival of breast‐cancer‐bearing mice [10, 11, 12].

We adopted TP‐Tox as an established bifunctional peptide with anti‐cancer activity. In the course of the study, we discovered another peptide conjugate TP2‐Tox, which was derived from TP‐Tox, demonstrating toxicity towards several solid tumor cell lines. By generating another derivative from TP‐Tox, called S2‐Tox as a less toxic control, we aimed to characterize the recently discovered toxic derivative of the TP‐Tox bifunctional peptide.

## 2 Materials and Methods

### 2.1 Cell Lines

All cell lines investigated in this study are described in Table S1.

### 2.2 Bifunctional Peptide Design and Synthesis

TP‐Tox (LTVSPWY‐GG‐(KLAKLAK)2), TP2‐Tox (VYSPWLT‐GG‐(KLAKLAK)2) and S2‐Tox (LTYQTWP‐GG‐(KLAKLAK)2) were designed by coupling the targeting domain to the toxic (KLAKLAK)2 domain through a glycine bridge, as previously described [12]. All peptides were synthesized, purified using HPLC, and confirmed with mass spectrometry by Eurogentec (Seraing, Belgium). The *D‐*Tox and FLAG conjugates of all peptides were also manufactured by Eurogentec. The FITC‐conjugated peptides were synthesized by Thermo Fisher Scientific. All peptides were reconstituted in sterile distilled water.

### 2.3 Measuring Peptides Toxicity Towards the Cancer Cell Lines

7 × 10^3^–10^4^ cells at 80%–90% confluency were seeded in a 96‐well plate. After 24 h, the cells were treated with peptides at different concentrations for another 24 h. The treatment was discontinued by changing the medium. The viability of the cells was measured colorimetrically by incubating the cells with cell‐counting kit 8 reagent (Dojindo, Kumamoto, Japan) for 30 min–2 h until significant color developed. The absorbance was measured at 450 and 620 nm with Multiskan FC (Thermo Fisher Scientific).

### 2.4 Measuring the Peptides Uptake Rate

7 × 10^3^–10^4^ cells were seeded in a 96‐well plate. On the next day, the cells were incubated with 5 μM of FITC‐conjugated peptides. The cells were harvested at different time points and measured for their fluorescence intensity using BD FACSCanto II (Becton, Dickinson and Company, Franklin Lanes, NJ, USA). Dead cells were excluded by staining the cells with 7‐AAD dye (Biolegend, San Diego, CA, USA).

### 2.5 Co‐Immunoprecipitation Assay and Mass Spectrometry Analysis

Cells were grown to reach near confluency in T‐75 flasks and lysed using a detergent‐based buffer supplemented with a proteinase inhibitor (Thermo Fisher Scientific). The lysate was incubated with peptide conjugated to the FLAG sequence (Eurogentec) on a rotator at room temperature for 1 h. To isolate the protein‐peptide complexes, Dynabeads coupled to the anti‐FLAG antibody (Thermo Fisher Scientific) were resuspended in the lysate and incubated on a rotator for 30 min at 4°C. Further isolation and purification of the protein complexes were performed according to the manufacturer's instructions (Thermo Fisher Scientific).

The isolate was sent to be analyzed by mass spectrometry in the Core Facility of Analytical Proteomics at the University of Bonn, Germany.

### 2.6 Western Blot

Cells were grown to reach near confluency in a T‐75 flask. The cells were lysed using RIPA buffer (Thermo Fisher Scientific) supplemented with a proteinase inhibitor. Cell debris was removed from the lysate by centrifugation. The protein concentration of the lysate was measured using Bradford reagent (Thermo Fisher Scientific).

Each sample containing 20 μg of protein was loaded onto handcast SDS‐Polyacrylamide gel with 12% resolving gel and run at 80–100 V in Laemmli Buffer (Serva, Heidelberg, Germany). The gel was immediately transferred onto a nitrocellulose membrane using wet transfer at 90 V for 90 min. The blot was stored in blocking buffer (Bio‐Rad Laboratories Inc., Hercules, CA, USA) overnight at 4°C. The immunostaining was started by staining the blot with primary antibody on a shaker for 2 h at room temperature. The remaining staining with the secondary antibody and colorimetric reagents was performed using amplified Opti‐4CN substrate kit (Bio‐Rad Laboratories Inc.) per manufacturer's manual.

### 2.7 Knockdown Establishment and Verification

Knockdown of the target genes was performed by introducing DsiRNA (Table S2) into the cells using Lipofectamine RNAiMAX (Thermo Fisher Scientific) as instructed by the manufacturer.

The gene knockdown was verified by qPCR using the QuantStudio 3 Real‐Time PCR System (Thermo Fisher Scientific). The RNA of the knockdown cells was isolated using the RNeasy Mini Kit (Qiagen, Hilden, Germany) per the manufacturer's instruction and analyzed using NanoDrop (Thermo Fisher Scientific). The qPCR mix was prepared using TaqPath 1‐Step Multiplex Master Mix (Thermo Fisher Scientific) and Taqman probes (Thermo Fisher Scientific): Hs00610058_m1, Hs05045008_s1, Hs02597812_g1, Hs00356629_g1, Hs03834446_m1, Hs00853081_g1, Hs05057028_s1, and Hs02786624_g1.

### 2.8 Checking the Peptide Toxicity Towards the Knockdown Cells

After 48 h of transfection with DsiRNA, the cells were harvested and seeded at 7 × 10^3^–10^4^ cells per well in a 96‐well plate and left overnight to adhere to the well. 8 μM of peptides was added onto the cells, and at the end of the treatment, the viability of the cells was measured colorimetrically using the cell‐counting kit 8 reagent.

To check the peptide uptake rate of the knockdown, the cells were treated with 5 μM of FITC‐conjugated peptide. The cells were then harvested and the fluorescence intensity of the cells was measured using flow cytometry BD FACSCanto II. Dead cells were excluded by staining the cells with 7‐AAD.

### 2.9 Detecting Plasma Membrane Damage

7 × 10^3^–10^4^ cells were seeded in a 96‐well plate and left to recover overnight. The cells were treated with 8 μM of peptides and were harvested at two time points. The treated cells were harvested, and the supernatants were collected.

The cells were stained with FITC Annexin V and 7‐AAD (Biolegend) in Annexin V binding buffer (Becton, Dickinson and Company). The binding of Annexin V was checked using flow cytometry.

25 μL of the supernatant was used to check the presence of active lactate dehydrogenase using the CyQUANT LDH Cytotoxicity Assay Kit (Thermo Fisher Scientific) according to the manufacturer's instructions. The absorbance was measured at 490 and 680 nm.

### 2.10 Colocalization Assay

2 × 10^4^ cells were seeded on a gelatin‐coated coverslips in a 24‐well plate and left to recover. The cells were incubated with 3 μM of FITC‐peptide for 2 h and fixed with 4% paraformaldehyde. The fixed cells were stained with MitoTracker Red (Thermo Fisher Scientific) for 20 min at 37°C. The stained coverslips were counterstained with Hoechst‐33258 for 5 min and carefully mounted on an object glass using Aqua‐Poly/Mount (Polysciences Inc., Warrington, PA, USA). The mounted samples were left to solidify overnight. The slides were analyzed using a Leica SP8 confocal microscope.

### 2.11 Caspase Activity Assay

1.5–2 × 10^4^ cells were seeded in a 48‐well plate and left to recover overnight. The cells were then treated with 8 μM of peptides and 80–100 μM of CCCP as the positive control. After the treatment, the cells were harvested and incubated with CellEvent Caspase 3/7 detection reagent (Thermo Fisher Scientific) or Caspase 9 (active) staining kit (Abcam Inc., Waltham, MA, USA) for 30 min–1 h at 37°C. To exclude the dead cells, the cells were stained with 7‐AAD. The fluorescence signal was measured by flow cytometry.

### 2.12 Inhibiting Caspase Activity

7 × 10^3^–10^4^ cells were seeded in a 96‐well plate and left to recover overnight. The cells were pre‐incubated with 40 μM of z‐VAD‐FMK (Medchemexpress, Monmouth Junction, NJ, USA) for 2 h at 37°C. 8 μM of peptides were added to the pretreated cells, and the cells were incubated for 16 h. Viability of the cells was analyzed using the cell‐counting kit 8 reagent.

### 2.13 Measuring Calpain Activity

7 × 10^3^–10^4^ cells were seeded in a 96‐well plate and left to recover overnight. The cells were incubated with 8 μM of peptides and harvested at the end of the treatment. CMAC and *t*‐BOC‐Leu‐Met substrate (Thermo Fisher Scientific) were added onto the cells and incubated at 37°C for 30 min. The cells were stained with 7‐AAD to exclude the dead cells. The fluorescence signal was measured by flow cytometry.

### 2.14 Detecting MLKL Pore Formation

30–40 × 10^4^ cells were seeded on gelatin‐coated cover slips in a 24‐well plate and left to recover overnight. The cells were treated with 8 μM of peptides. After treatment, the cells were fixed with 4% paraformaldehyde (Thermo Fisher Scientific) and permeabilized with 2% Triton X 100 (Carl Roth GmbH, Karlsruhe, Germany). The cells were blocked with 5% goat serum and stained with MLKL monoclonal antibody (Proteintech, Rosemont, IL, USA) and ErbB2 polyclonal antibody (Proteintech) for 1 h at room temperature. The secondary staining was performed by incubating the cells with anti‐mouse Alexa Fluor647 antibody (Cell Signaling Technology Inc., Danvers, MA, USA) and multi‐rAb goat anti‐rabbit CoraLitePlus 488 antibody (Proteintech) for 30 min at room temperature. The counterstain was done by incubating the cells with DAPI (Merck KgaA, Darmstadt, Germany) for 5 min. The coverslips were carefully mounted to the object glass using Aqua‐Poly/Mount and left at room temperature overnight to solidify. The slides were analyzed using a Leica SP8 confocal microscope in the Microscopy Core Facility, University of Bonn, Germany.

### 2.15 Data Analysis

The flow cytometry data was analyzed with FlowJo V10.10.0 software (Becton, Dickinson and Company). The microscopy pictures were analyzed using Fiji software.

All statistical analysis was performed in Graphpad Prism V10 software (Graphpad Software, San Diego, CA, USA). Multiple comparisons were done using ordinary one‐way ANOVA with post comparison to the untreated group by Bonferroni's test. Data are represented as mean ± SEM. *p < 0.05, **p < 0.01, and ***p < 0.001.

## 3 Results

### 3.1 Enhanced Toxicity of the Peptide Conjugates Was Correlated With Their Penetration Into the Target Cells

**FIGURE 1:**
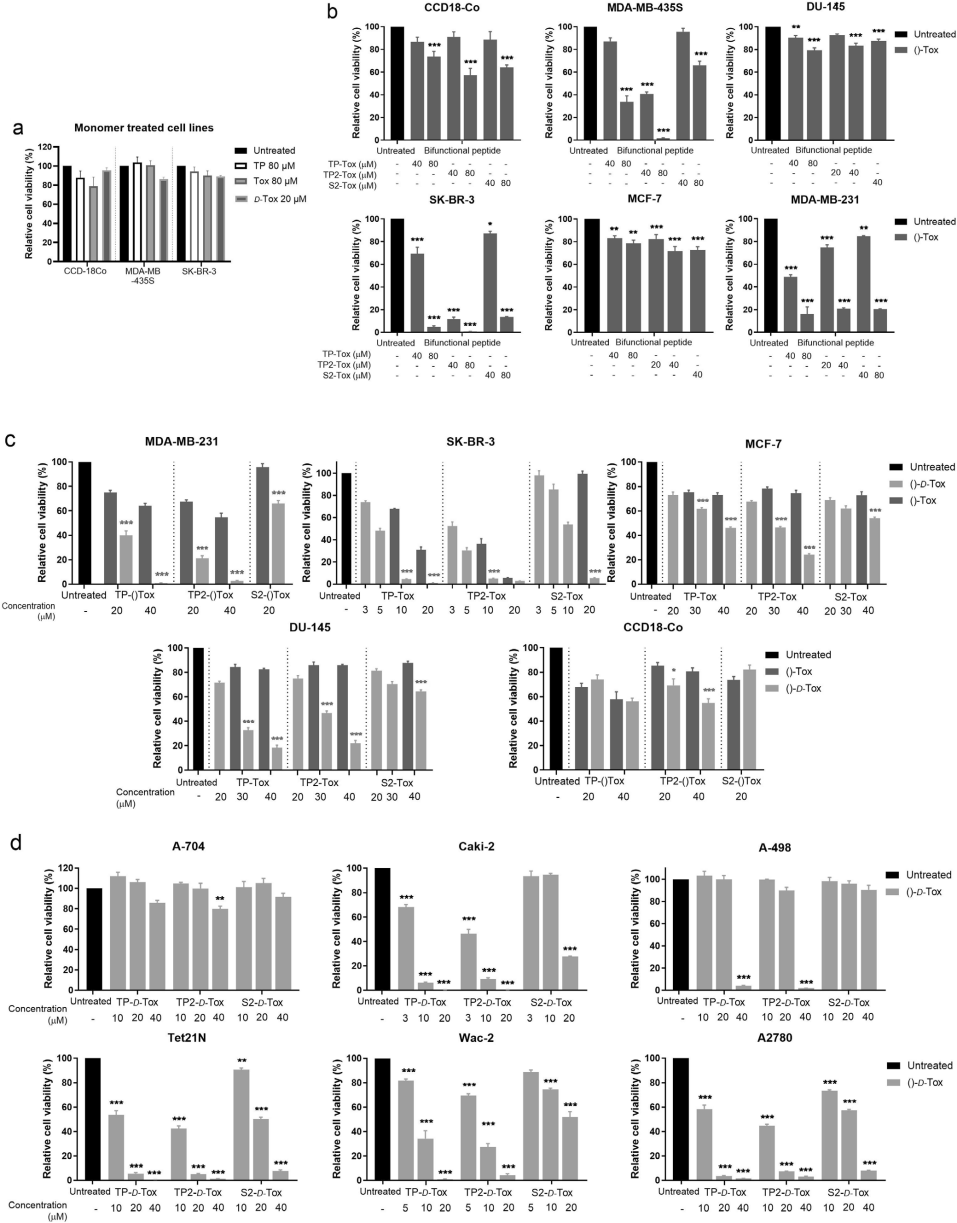
Inhibiting cancer cell lines with bifunctional peptides. Cell lines were treated for 24 h with (a) Peptide monomers TP, Tox, and *D*‐Tox, (b) TP‐Tox, TP2‐Tox and S2‐Tox peptides, (c) All peptide conjugates in ‐Tox and –*D*‐Tox conformation, (d) TP‐*D*‐Tox, S1‐*D*‐Tox and S2‐*D*‐Tox. Data are presented as means of relative viability percentage ± SEM calculated by referring to untreated samples as 100% (*n* = 3–9). All statistical analysis was calculated using Bonferonni's one‐way ANOVA test. *p < 0.05; **p < 0.01; ***p < 0.001 showed the differences against untreated samples, while *p < 0.05; **p < 0.01; ***p < 0.001 determined the differences between the ()‐Tox treated and its particular ()‐*D*‐Tox treated group.

Coinciding with the previous study, TP‐Tox significantly reduced the number of living cells, while the TP and Tox peptides did not cause a noticeable effect on the cell lines (Figure 1a,b). Surprisingly, TP2‐Tox, which was designed by scrambling the targeting sequence of TP‐Tox, achieved similar anti‐tumor activities at roughly half of the TP‐Tox concentration. On the other hand, the scrambled peptide S2‐Tox caused considerable cell death of the cell lines only at fairly higher concentrations.

Substituting the ‐Tox sequence with –*D‐*Tox greatly reduced the IC_50_ of all peptides for all cell lines apart from CCD‐18Co (Figure 1c). Preferring the *D*‐isomer for constructing (KLAKLAK)2 has been previously tested. The *D*‐(KLAKLAK)2 induced the same effects as its mirrored counterpart, which was possible due to the reciprocal chiral specificity towards their target molecules. Selecting the *D*‐enantiomer was reported to enable the corresponding peptide to gain a higher resistance towards proteolysis and to improve its half‐life in vivo. Despite the higher cost to synthesize *D*‐amino acids, the *D*‐peptide showed potential to have a higher physiological activity when compared to its stereoisomer [4, 13, 14].

**FIGURE 2:**
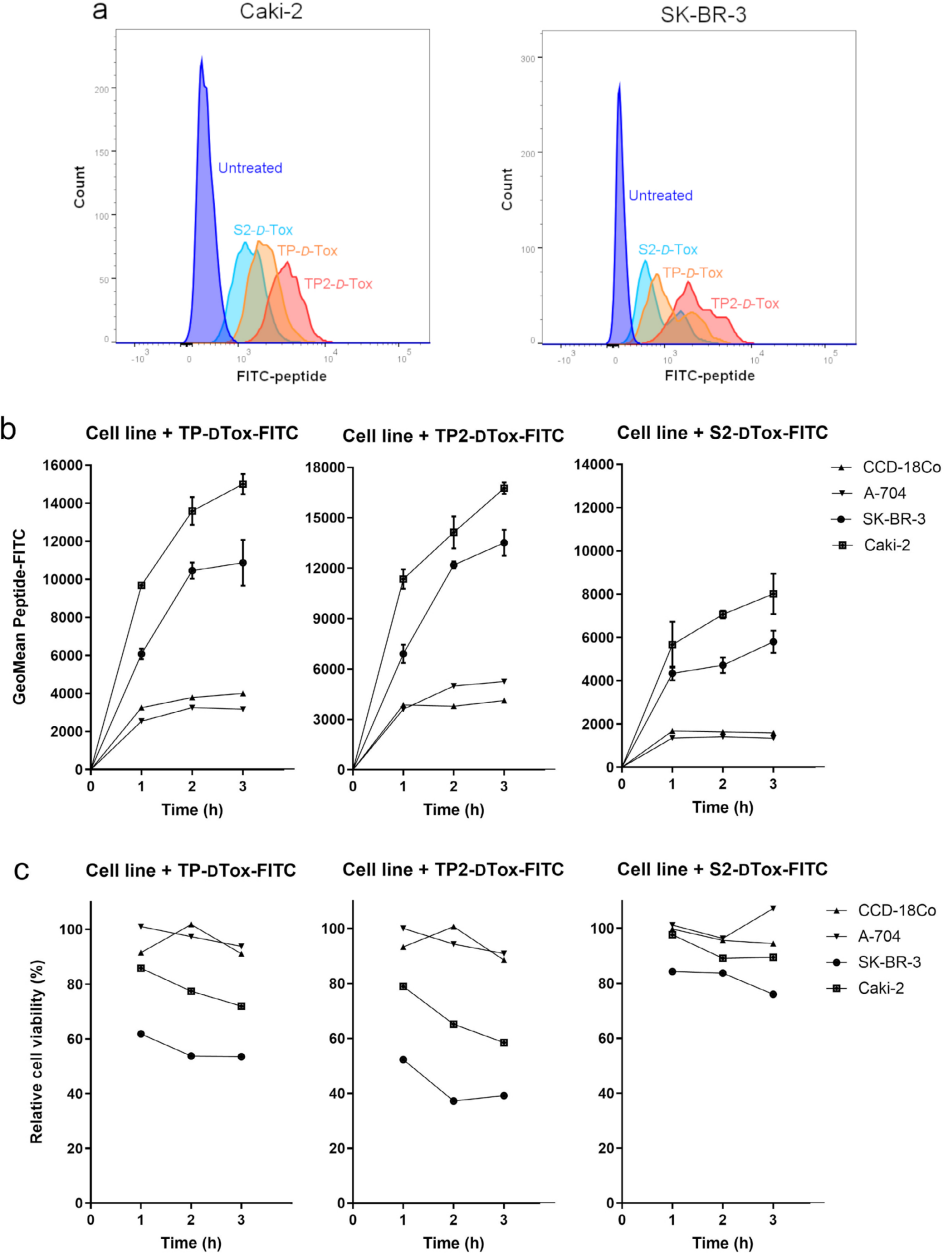
Determining internalization rate of the different peptides into the cells by: (a) measuring the fluorescence intensity of Caki‐2 and SK‐BR‐3 1 h after the addition of the FITC‐peptides, (b) adding FITC‐peptides to Caki‐2, SK‐BR‐3, A‐704 and CCD18‐Co and measuring the fluorescence intensity of the cells at different time points, (c) measuring the viability of the cells after peptide treatment at certain time points. Some data are presented as mean ± SEM (*n* = 1–3). The cell viability was calculated by normalizing the treated samples to untreated samples.

We further tested the *D*‐Tox peptides in renal carcinoma, neuroblastoma, and ovarian carcinoma cell lines (Figure 1d). From all cell lines we tested, a higher toxic activity of TP2‐*D*‐Tox was detected for MDA‐MB‐435S, MDA‐MB‐231, SK‐BR‐3, Caki‐2, Tet21N, Wac‐2, and A2780 cell lines. The increased susceptibility towards the TP2‐*D*‐Tox correlated with the higher susceptibility towards TP‐ and S2‐*D*‐Tox peptide. This finding indicated that TP2‐*D*‐Tox interacted with the same target molecules as TP‐ and S2‐*D*‐Tox but with differing affinities. Therefore, to characterize TP2‐*D*‐Tox mechanism, we used S2‐*D*‐Tox as the less toxic control and TP‐*D*‐Tox as the established peptide control. Measuring the internalization of the three peptide conjugates into the cells revealed that a higher cell penetration contributed to TP2‐*D*‐Tox's superior toxicity (Figure 2a).

To further confirm the relation between cell penetration and peptide toxicity, we classified the tested cell lines as peptide‐susceptible, including SK‐BR‐3 and Caki‐2, and peptide‐resistant, consisting of A‐704 and CCD‐18Co. By treating the cells with FITC‐conjugates of ‐*D*‐Tox, we observed that the peptide‐resistant cells had a much lower FITC signal gain when compared to the peptide‐susceptible cells during the first 3 h of peptide treatment (Figure 2b). The peptide infiltration into the cells was shown to negatively correlate with the viability of the cells independent of their susceptibility (Figure 2c). These data suggested that the intracellular presence of the peptides was one of the prerequisites for their toxic activities.

### 3.2 The Peptide Binding to NMIIA's Myosin Light Chains and S100A9 Triggered the Internalization of the Peptide via Clathrin‐Mediated Endocytosis

**FIGURE 3:**
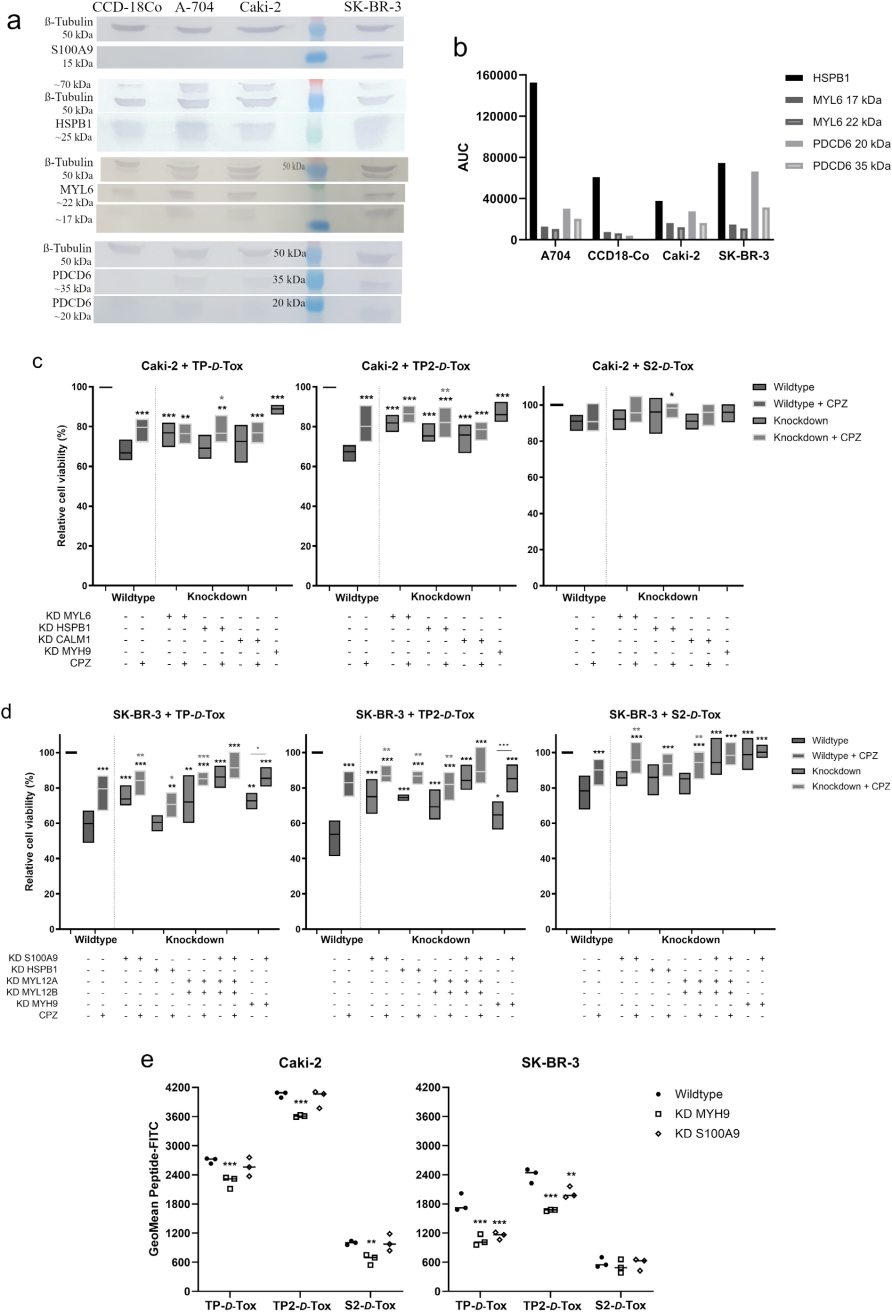
Assessing the peptide escort candidates by (a) measuring the protein expression using western blot and (b) normalizing the detected protein bands to ß‐tubulin, (c) knocking down the respective genes and checking the effect in cell viability after 1–2 h of peptide treatment in the presence or absence of the endocytosis inhibitor in Caki‐2 or (d) SK‐BR‐3 cell line, (e) The effects of knocking down the genes on the internalization of the FITC‐peptide conjugates after 1 h of treatment. Data of c, d are presented as means of the relative viability ± SEM calculated from normalizing the treated samples to the untreated cell line (*n* = 3–9). *p < 0.05; **p < 0.01; ***p < 0.001 difference was calculated using Bonferroni's test between peptide treated samples and untreated samples. *p < 0.05; **p < 0.01; ***p < 0.001 determined the differences between knockdown and its corresponding CPZ treated group. Data of e are displayed as means ± SEM, *p < 0.05; **p < 0.01; ***p < 0.001 difference was calculated using Bonferroni's test between the knockdown and wild‐type groups.

To determine the ligand for TP2‐*D*‐Tox, which was responsible for facilitating cell entry, we tagged TP2‐ and S2‐*D*‐Tox with FLAG. The FLAG‐peptides were allowed to interact with the proteins in the cell lysates from Caki‐2 and SK‐BR‐3. The resulting peptide‐protein complexes were isolated using anti‐FLAG antibody and identified by mass spectrometry (Tables 1 and 2). We selected the ligand candidates by their high affinity for TP2‐ rather than S2‐*D*‐Tox and their presence in the plasma membrane to include the interactions, which were likely to occur under biological conditions. By evaluating their basal expression between the peptide‐resistant and ‐susceptible cell lines, we found that only S100A9 was noticeably expressed in SK‐BR‐3 (Figure 3a). Neither PDCD6, MYL6, nor HSPB1 were specifically present or absent in the peptide‐resistant or ‐susceptible cell lines (Figure 3a,b).

**TABLE 1:** List of proteins in the Caki‐2 lysate, which were isolated using the FLAG‐peptides. The binding of the peptides to the proteins in the cell lysate was limited by NaCl concentrations of 100 and 150 mM. The reading for each protein is normalized to the total protein signal. The proteins selected for further analysis are marked by italicizing the corresponding genes.

Gene symbol	TP2‐*D*‐Tox‐DYKDDDDK fraction		S2‐*D*‐Tox‐DYKDDDDK fraction		Abundance ratio (TP2/S2)	
	100 mM	150 mM	100 mM	150 mM	100 mM	150 mM
NPM1	3.2E+07	6.0E+06	Not found	Not found	TP2 fraction	TP2 fraction
PCMT1	1.4E+06	9.2E+06	Not found	Not found	TP2 fraction	TP2 fraction
*PDCD6*	1.3E+08	1.1E+09	Not found	1.2E+08	TP2 fraction	9.3
LGALS1	2.0E+07	4.6E+07	Not found	4.2E+06	TP2 fraction	10.8
TUBB	1.2E+07	3.4E+07	Not found	1.3E+07	TP2 fraction	2.7
DYNLRB1	4.3E+07	2.2E+07	Not found	5.1E+06	TP2 fraction	4.3
PFN1	4.0E+06	1.5E+07	Not found	2.0E+06	TP2 fraction	7.3
FASN	1.8E+06	1.8E+07	Not found	4.0E+06	TP2 fraction	4.4
S100A7	1.8E+06	1.7E+07	Not found	4.7E+07	TP2 fraction	0.4
SKP1	5.9E+06	1.7E+06	Not found	2.4E+07	TP2 fraction	0.1
*HSPB1*	6.5E+07	8.5E+07	7.1E+05	9.4E+06	91.8	9.1
*MYL6*	2.8E+07	2.7E+07	6.9E+05	3.8E+08	41.0	0.1
PRDX1	1.2E+07	1.1E+07	5.6E+05	3.0E+06	21.5	3.7
TUBA1A	1.8E+07	2.8E+07	1.0E+06	1.4E+07	17.9	2.0
RPS28	1.4E+09	8.8E+08	7.8E+07	6.6E+07	17.5	13.4
RPL4	2.0E+07	5.0E+06	1.2E+06	9.2E+06	16.2	0.5
NAP1L1	5.9E+06	5.4E+06	3.8E+05	Not found	15.5	TP2 fraction
ACTG2	3.8E+06	1.1E+06	3.4E+05	3.3E+05	11.3	3.4
TXN	1.3E+07	1.5E+07	1.4E+06	5.4E+06	9.5	2.7
*S100A9*	1.2E+07	1.1E+07	1.4E+06	5.4E+07	8.5	0.2
DSP	7.8E+06	1.4E+06	1.4E+06	3.8E+06	5.7	0.4
ACTG1	1.0E+07	1.5E+07	1.9E+06	1.3E+07	5.6	1.1
JUP	6.6E+06	1.7E+06	2.1E+06	5.5E+06	3.1	0.3
*MYH9*	9.0E+05	5.7E+06	4.3E+05	2.2E+06	2.1	2.7
LGALS7	1.2E+06	1.2E+06	1.1E+06	5.3E+06	1.1	0.2
COL1A1	4.0E+07	5.4E+06	4.3E+07	1.1E+06	0.9	5.2
SNCA	1.4E+07	8.8E+06	4.6E+07	8.9E+06	0.3	1.0
RPLP2	8.9E+08	1.5E+09	7.1E+09	2.8E+09	0.1	0.6
*CALM1*	2.0E+06	1.0E+07	1.7E+07	8.2E+06	0.1	1.3
RPLP1	8.1E+06	1.1E+08	6.1E+08	8.9E+07	0.0	1.2

**TABLE 2:** List of proteins in the SK‐BR‐3 lysate, which were isolated by the FLAG‐peptides. The binding of the peptides to the proteins in the cell lysate was limited by NaCl concentrations of 100 and 150 mM. The reading for each protein is normalized to the total protein signal. The proteins selected for further analysis are marked by italicizing the corresponding genes.

Gene symbol	TP2‐*D*‐Tox‐DYKDDDDK fraction		S2‐*D*‐Tox‐DYKDDDDK fraction		Abundance ratio (TP2/S2)	
	100 mM	150 mM	100 mM	150 mM	100 mM	150 mM
BANF1	1.0E+08	3.1E+05	Not found	Not found	TP2 fraction	TP2 fraction
DDX1	2.9E+07	Not found	Not found	Not found	TP2 fraction	Not found
*PDCD6*	5.3E+07	1.1E+07	Not found	Not found	TP2 fraction	TP2 fraction
SKP1	4.2E+07	4.6E+05	Not found	2.7E+06	TP2 fraction	0.17
CLTA	1.8E+07	Not found	Not found	Not found	TP2 fraction	Not found
EIF6	6.3E+06	9.3E+07	Not found	3.6E+07	TP2 fraction	2.61
TUBB	5.4E+06	Not found	Not found	Not found	TP2 fraction	TP2 fraction
SUSD2	1.0E+06	2.6E+06	Not found	Not found	TP2 fraction	TP2 fraction
*MYL6*	5.6E+08	5.2E+06	8.5E+05	3.6E+06	660.13	1.46
*HSPB1*	6.4E+07	9.1E+06	3.2E+05	Not found	200.20	TP2 fraction
*MYL12B*	3.6E+07	Not found	1.9E+05	Not found	185.90	Not found
*MYL12A*	3.6E+07	Not found	1.9E+05	Not found	185.90	Not found
RTRAF	5.1E+07	8.9E+04	4.9E+05	Not found	105.35	TP2 fraction
PCBP1	1.0E+07	1.6E+06	2.1E+05	9.7E+04	48.72	16.66
RPS28	4.3E+08	4.8E+08	5.9E+07	4.9E+07	7.37	9.81
RPSA	1.1E+07	2.8E+06	1.6E+06	Not found	6.67	TP2 fraction
RPLP12	3.7E+07	1.9E+05	5.9E+06	8.8E+05	6.32	0.21
RPS20	1.9E+07	9.5E+04	3.7E+06	1.2E+05	5.20	0.82
*S100A9*	2.5E+07	7.2E+06	5.5E+06	5.3E+06	4.54	1.37
*CALM1*	3.1E+07	6.6E+07	7.3E+06	2.0E+07	4.23	3.32
ACTG1	1.6E+07	4.1E+06	7.1E+06	2.9E+06	2.32	1.38
CALML5	4.5E+06	4.5E+06	2.0E+06	6.4E+06	2.21	0.70
DCD	2.8E+07	2.0E+07	1.4E+07	1.5E+07	2.06	1.37
CSTA	2.9E+06	6.0E+06	2.0E+06	2.8E+06	1.46	2.18
RPS21	3.8E+07	6.4E+06	3.0E+07	2.8E+06	1.26	2.31
RPLP1	5.5E+06	2.9E+09	3.5E+08	1.3E+09	0.02	2.26

Therefore, we performed functional assays to reevaluate the proteins by knocking down the target genes to check how the peptide toxicity was affected (Figure S1a). The lethal activity of TP2‐*D*‐Tox was significantly restricted in Caki‐2 when MYL6, HSPB1, or CALM1 expression was inhibited, while TP‐*D*‐Tox activity was reduced only in MYL6 knockdown cells (Figure S1b). Similarly, in SK‐BR‐3, inhibiting HSPB1 expression restricted the toxicity of TP2‐ but not TP‐*D*‐Tox. In addition, inhibiting S100A9, MYL12A, MYL12B expression hampered the toxic effect of TP‐ and TP2‐*D*‐Tox, while knocking down MYL6, CALM1, and PDCD6 raised the toxicity of all peptides (Figure S1c).

TP‐Tox was previously suggested to enter the cells via endocytosis upon binding to G3BP1 [12, 15]. The presence of actin, clathrin A, tubulin, and calmodulin in the isolated peptide‐protein complexes supported the model that TP2‐*D*‐Tox also penetrated the cells via the endosomal pathway (Tables 1 and 2). As TP‐ and TP2‐*D*‐Tox peptides were shown to share affinities with some ligands, we investigated whether clathrin‐mediated endocytosis (CME) was involved in escorting TP2‐*D*‐Tox into the cells by including chlorpromazine (CPZ) during the peptide treatment. CPZ temporarily anchored clathrin and its adaptor protein to intracellular vesicles, hampering clathrin‐coated pit formation [16]. We found that the addition of CPZ notably lowered the toxic effect of all peptides for Caki‐2 and SK‐BR‐3 (Figure 3c,d). Furthermore, concurrent knockdowns of the investigated genes increased cell resistance towards peptide toxicity compared to the single gene knockdown (Figure S1d). Based on the evident interactions, the ligands of TP2‐*D*‐Tox were possibly interrelated subunits of a multimeric structure, which was diversely expressed in one cell type.

Non‐muscle myosin II (NMII) is a multimeric protein consisting of heavy chains, regulatory (RLC) and essential light chains (ELC), which are expressed in NMIIA, IIB, and IIC isoforms encoded by MYH9, MYH10, and MYH14, respectively [17, 18, 19]. In the context of endosomal trafficking, the majority of the proteins found to correlate with the TP‐ and TP2‐*D*‐Tox toxicity can be described as subunits of NMII. MYL6, MYL12A, and MYL12B were previously depicted to serve as RLC and ELC of NMII. CALM1 can bind to the neck domain of NMII and, upon activation by elevated intracellular calcium, it activates myosin light chain kinases, leading to the phosphorylation of RLCs [18, 20, 21].

One of the heavy chains of the NMII isoform, NMIIA encoded by the MYH 9 gene, was isolated by both TP2‐ and S2‐*D*‐Tox from Caki‐2 lysate (Table 1). We confirmed that the presence of NMIIA was important in maintaining the toxicity of the peptides towards Caki‐2 and SK‐BR‐3, although the NMIIA heavy chain was not captured by the peptides from SK‐BR‐3 lysate (Figure 3c,d, Table 2). Further analysis revealed that the peptides toxicity towards SK‐BR‐3 did not only depend on NMIIA, but also on S100A9. Moreover, we observed no noticeable viability improvements when the CME route of the simultaneous knockdown of MYH9 and S100A9 was blocked. This result indicated that the peptide internalization route via CME in SK‐BR‐3 was initiated by binding to S100A9 and NMIIA's RLC, MYL12A and MYL12B (Figure 3d).

However, this significance of S100A9 on the peptide toxicity towards SK‐BR‐3 was not observed in Caki‐2, likely due to the low expression of S100A9, as detected by both mass spectrometry and western blot (Table 1, Figure 3a). Hence, unlike in SK‐BR‐3, the maximum inhibition of the peptides toxicity in Caki‐2 was achieved by decreasing NMIIA but not S100A9 (Figure 3c; Figure S1b). These results suggested that the peptides entry into Caki‐2 occurred by binding to the NMIIA's ELC, MYL6, leading to peptide internalization via CME.

Our data indicated that the peptide ligands were distinctly expressed by Caki‐2 and SK‐BR‐3. The affinity of the peptides towards MYL6 was confirmed for both Caki‐2 and SK‐BR‐3, as MYL6 from both cells was isolated by co‐immunoprecipitation (Tables 1 and 2). However, MYL6 was shown to only correlate with the peptide toxicity in Caki‐2, suggesting that under physiological conditions, the peptide was able to access MYL6 in Caki‐2 but not SK‐BR‐3 (Figure S1c). On the other hand, MYL12A and MYL12B were demonstrated as the ligands of TP‐ and TP2‐*D*‐Tox only in SK‐BR‐3 (Tables 1 and 2). Although Caki‐2 expressed the transcript of both light chains, the proteins did not seem to bind to the peptide during co‐immunoprecipitation (Figure S2a, Table 1). This discrepancy of peptide binding suggested that the epitope preferred by the peptides was only present in MYL12A and MYL12B of SK‐BR‐3.

As subunits of the non‐muscle myosin complex, diversification of myosin light chains has been previously reported to support the distinct activities of the cells. Many species‐ or tissue‐specific isoforms were identified for muscle and non‐muscle myosin molecules. Protein diversification can be established from mutation, post‐translational modification, alternate splicing, or gene duplication. Alternate splicing of ELC and RLC genes was described to enrich the light chain repertoire at the protein level, altering the expression level of these proteins. The variation that emerged in the NM isoforms was shown to partly associate with a disease or an age‐related state [17, 22].

### 3.3 HSPB1 Demonstrated a Protective Effect Towards TP2‐*D*‐Tox Toxicity

HSPB1 was shown to correlate with TP2‐*D*‐Tox toxicity towards Caki‐2 and SK‐BR‐3 (Figure 3c,d). HSPB1 protein level has been reported to be upregulated upon diverse cellular stress and contribute significantly to maintaining proteostasis [23, 24, 25].

HSPB1 was reported to be overexpressed in many types of cancers, promoting tumorigenesis and resistance against many anticancer drugs and host immune responses [24, 26, 27]. Our data revealed a stable HSPB1‐TP2‐*D*‐Tox interaction with a high affinity. Interestingly, HSPB1 depletion significantly impaired TP2‐*D*‐Tox toxicity, without affecting TP‐ or S2‐*D*‐Tox. HSPB1 might have exerted its chaperone function on the suitable cytosolic peptides, protecting them from immediate proteolytic degradation.

**FIGURE 4:**
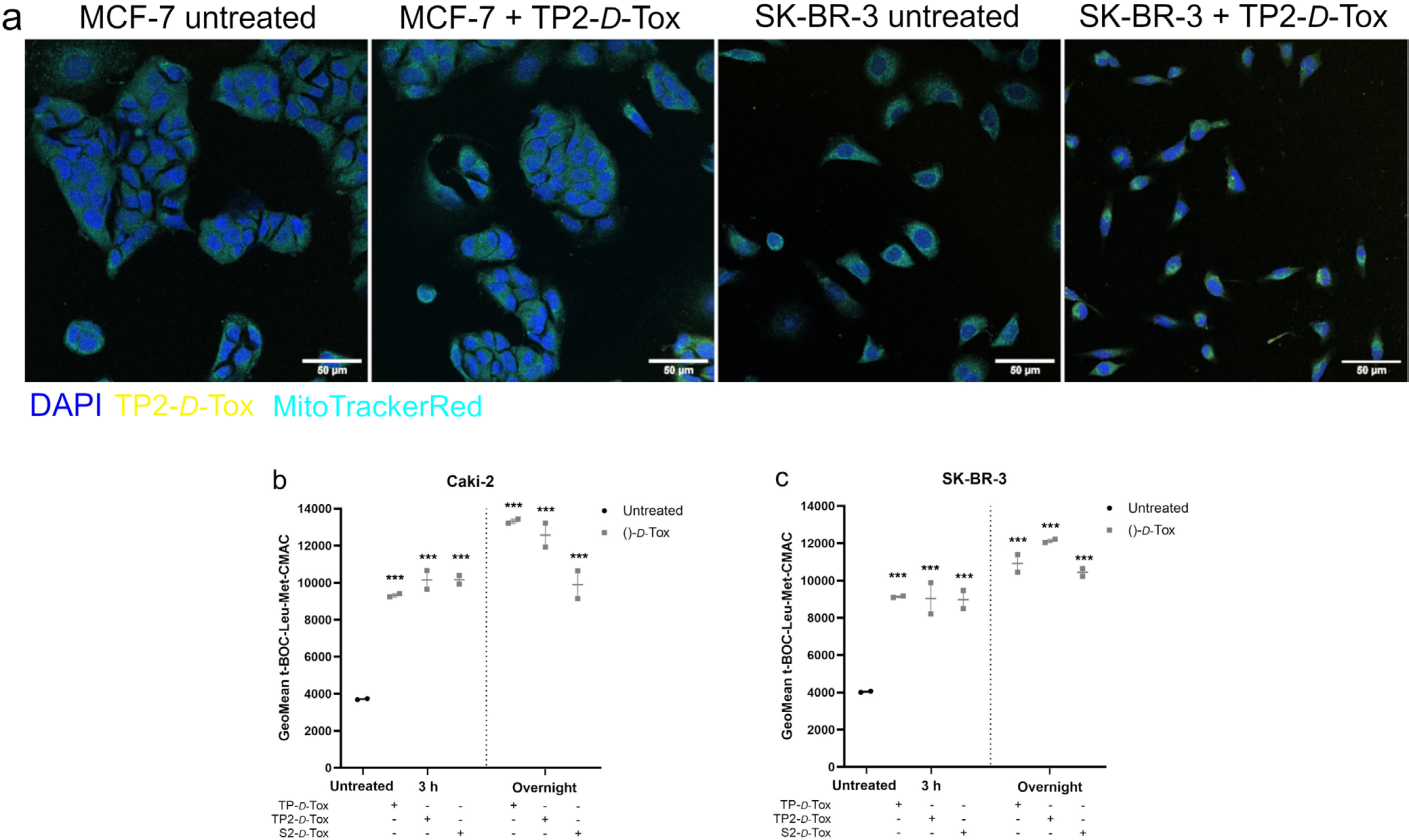
Evaluating the effect of peptide conjugate penetration into the cells by (a) checking the TP2‐ *D*‐Tox‐FITC colocalization with mitochondria in SK‐BR‐3 and MCF‐7 cell lines, measuring the activity of calpain in (b) Caki‐2 and (c) SK‐BR‐3. All measurements were performed at two time points after peptide treatment. Colocalization coefficients were calculated using ImageJ. Data points of b, c are shown as mean of geometric mean intensity ± SEM. *p < 0.05; **p < 0.01; ***p < 0.001 difference was calculated using Bonferroni's ANOVA one way test between peptide treated and untreated samples.

A recent study described that HSPB1 translocated to mitochondria following mild hyperthermia triggers [25]. As a binding partner of HSPB1, TP2‐*D*‐Tox was observed to partially colocalize with mitochondria in MCF‐7 and SK‐BR‐3 cells (Figure 4a; and Figure S4a,b), which were moderately and highly susceptible to the peptides, respectively. The colocalization coefficients indicated that TP2‐*D*‐Tox‐FITC overlapped mainly with mitochondria, while the excess of peptide was found in the cytoplasm. Analysis of SK‐BR‐3 quantified a lower M2 coefficient compared to MCF‐7, possibly due to a higher peptide penetration in SK‐BR‐3. Pearson's and Spearman's coefficients determined that there was a weak linear monotonic correlation between TP2‐*D*‐Tox‐FITC and the mitochondria, particularly in SK‐BR‐3. Overall, the correlation analysis suggested that inside the cells, TP2‐*D*‐Tox was transported to mitochondria, but when the peptide was intracellularly abundant, it was additionally distributed within the cytoplasm.

### 3.4 TP‐ and TP2‐*D*‐Tox Induced Distinctly Regulated Cell Death

The higher activity of calpain that was detected after treatment with the *D*‐Tox peptides implied that there was an increase of Ca^2+^ in the cytosol (Figure 4b,c). An increase in cytosolic Ca^2+^ was reported to cause an excessive accumulation of Ca^2+^ in the mitochondrial matrix via entry through the calcium uniporter, which led to a rapid release of mitochondrial calcium and proteins [28, 29, 30, 31].

**FIGURE 5:**
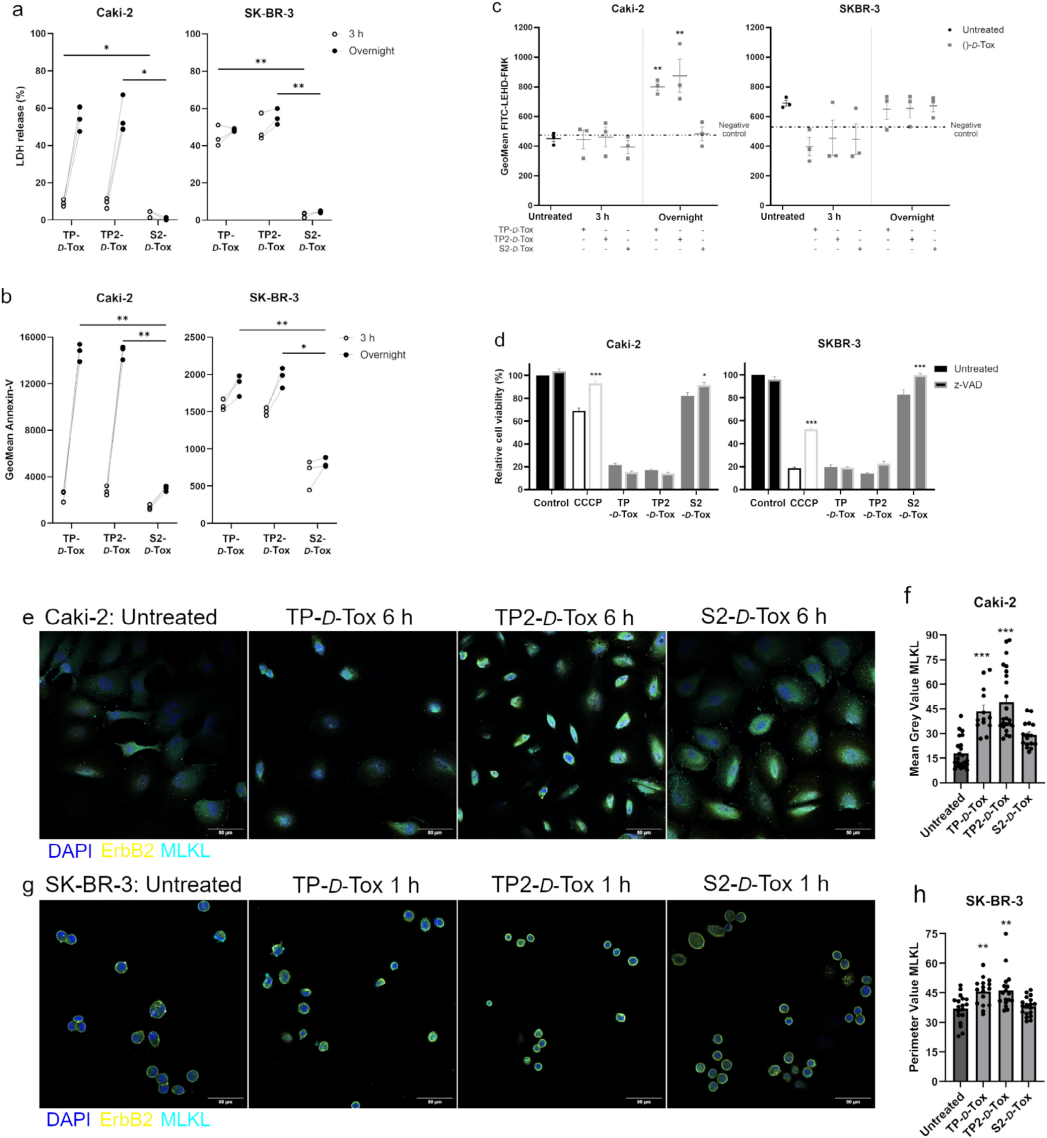
Analyzing cellular changes preceeding cell death due to the peptide treatment in Caki‐2 and SK‐BR‐3 by (a) detecting the LDH leakage, (b) measuring Annexin V binding, (c) detecting activity of caspases using LEHD‐FMK, (d) adding pan caspase inhibitor during the peptide treatment (*n* = 3), measuring the oligomerization of MLKL on the membrane of (e) Caki‐2 and (g) SK‐BR‐3. The signal of (e) and (g) was quantified and visualized in bar diagram as shown in (f) and (h). All analysis was performed when the cells were treated with the peptides. The data are depicted as mean ± SEM. Scale bar represents 50 μm. *p < 0.05; **p < 0.01; ***p < 0.001 difference was calculated using Bonferroni's ANOVA one way test between peptide treated and untreated sample.

A major interference in mitochondria functions might have caused oxidative phosphorylation to come to a halt. A preexisting mitochondrial dysfunction might have worsened the cellular energy crisis, impairing the ATPase function to maintain the gradient of Na^+^, Ca^2+^ and H^+^, leading to cytoplasmic acidification and ultimately, necrotic cell death [28, 29, 31]. We observed fast‐acting cell death processes with major plasma membrane damage detected within 3 h after TP‐ and TP2‐*D*‐Tox addition in SK‐BR‐3 (Figure 5a,b). The absence of caspase involvement in the development of peptide toxicity in SK‐BR‐3, while sustaining a severe mitochondrial dysregulation, suggested the possibility of undergoing necrosis (Figure 5c,d; and Figure S3b). The collapse of the SK‐BR‐3 plasma membrane was also indicated to manifest due to the translocation of MLKL oligomers (Figure 5g,h; and Figure S4d). In summary, the *D*‐Tox peptide penetration induced translocation of MLKL oligomers and elevation of cytosolic calcium, which might have resulted in mitochondrial dysfunction, weakening of the plasma membrane, and necrotic cell death in SK‐BR‐3.

Similar to SK‐BR‐3, upon foreign peptide infiltration, cytosolic calcium was elevated in Caki‐2. Considering that Caki‐2 cells did not experience major cell deaths within 6 h of peptide treatment, we assumed that the calcium increase might have assumed a minor contribution in inducing cell death, unlike in SK‐BR‐3. Although caspase activity was detected following TP‐ and TP2‐*D*‐Tox addition, neutralizing the activity with a pan‐caspase inhibitor did not affect the killing efficiency of the peptide. 6 h after peptide treatment, we observed the formation of MLKL oligomers on the plasma membrane of Caki‐2, which was especially intense when the cells were treated with TP‐ and TP2‐*D*‐Tox (Figure 5e,f; and Figure S4c). The lack of caspase involvement and membrane translocation of MLKL oligomers suggested that TP‐ and TP2‐*D*‐Tox induced necroptosis in Caki‐2.

## 4 Discussion

**FIGURE 6:**
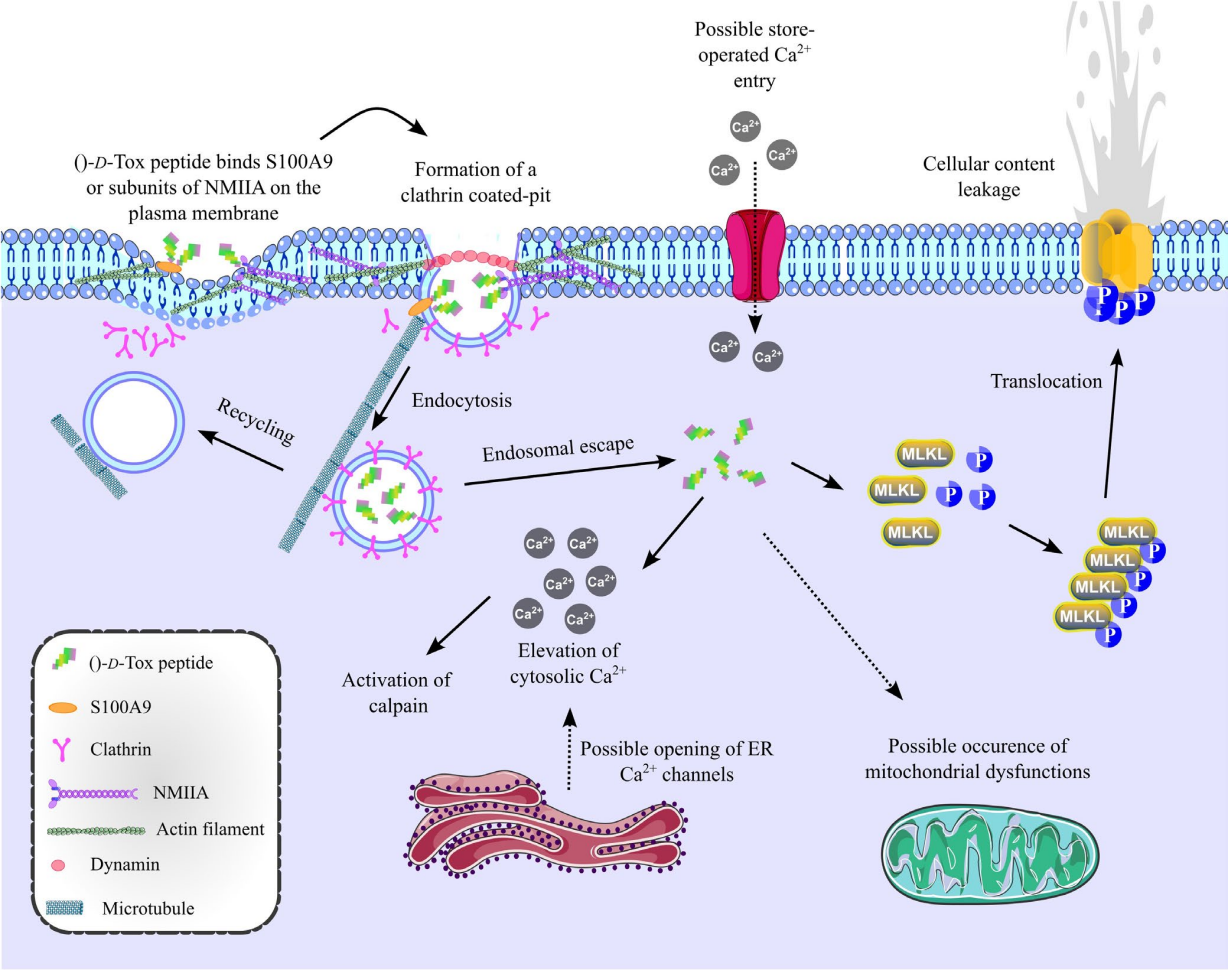
Schematic representation of ()‐*D*‐Tox peptide mode of actions. The schematic figure was designed using Inkscape V1.4.

We discovered that TP2‐*D*‐Tox peptide showed a higher toxicity towards Caki‐2 and SK‐BR‐3 compared to its parent peptide, TP‐*D*‐Tox. The peptides were shown to penetrate the cells via CME, triggered by binding to the myosin light chain of NMIIa and S100A9. The infiltration of the peptides caused mitochondrial dysfunction, leading to the accumulation of ROS and translocation of MLKL oligomers to the plasma membrane (Figure 6). The MLKL oligomers were shown to be mainly responsible for the plasma membrane collapse in Caki‐2. The rapid collapse of the plasma membrane in SK‐BR‐3 indicated that the peptide treatment might have induced necrosis derived from ATP insufficiency. The weakening of the plasma membrane and the formation of MLKL oligomers on the membrane led to plasma membrane rupture in SK‐BR‐3.

The higher intracellular *D*‐Tox peptides might have been recognized as distress warnings for invading pathogens or a large scale of unfolded proteins, which signaled the cells to initiate cell death. Necroptosis was reported to mediate the removal of bacteria in the phagocytosing immune cells or the invaded resident cells at the infection site to limit the area of infection and reduce the complementary tissue damage derived from excessive inflammation. The occurrence of unfolded protein peptides in the cytosol induces the activation of ER stress and may lead to cell death. Excessive activation of the unfolded protein response caused by an estrogen receptor inhibitor, BHPI, was observed to elevate cytosolic calcium, exhaust cellular ATP, trigger mitochondrial dysfunction, and ultimately cause necrotic cell death [32, 33].

Our work revealed the potential of TP2‐*D*‐Tox as an antitumor agent with a larger therapeutic index compared to TP‐*D*‐Tox. Since the ligands were previously shown to be overexpressed in many tumor types, we believe that by screening potential target cells based on their expression of the identified peptide ligands, more susceptible tumor entities toward this peptide might be discovered. Furthermore, NMIIA, S100A9, and HSPB1 upregulation in cancer has been correlated with the resistance of the targeted tumor to chemo‐, radio‐, and/or immunotherapy [34, 35, 36, 37]. Most therapeutic failures have been reported to emerge from the apoptosis resistance and evasion of the targeted tumor cells. To overcome this issue, many studies focus on developing approaches that trigger non‐apoptotic cell death [38]. Considering that TP2‐*D*‐Tox exerted its toxicity without activating any caspases, we think this peptide might be a promising therapeutic approach to bypass the caspase‐dependent apoptosis resistance issue in many tumors.

## Author Contributions

**Maria F. Setiawan:** conceptualization, data curation, formal analysis, investigation, methodology, project administration, validation, visualization, writing – original draft, writing – review and editing. **Oliver Rudan:** data curation, methodology, writing – review and editing. **Ingo G. H. Schmidt‐Wolf:** conceptualization, funding acquisition, supervision, writing – review and editing.

## Ethics Statement

The authors have nothing to report.

## Consent

The authors have nothing to report.

## Conflicts of Interest

The authors declare no conflicts of interest.

## Supporting information


**Figure S1.** Determining the escort protein candidates of the peptides.

**Figure S2.** Assessing mitochondrial membrane potential following the peptide treatment.

**Figure S3.** Measuring caspase 3 and 7 activity in the peptide‐treated cells.

**Figure S4.** Single channel figures from the immunostaining.

**Table S1.** Information about tested cell lines.

**Table S2.** List of DsiRNA utilized in the study.
