# Supplementary material for: Targeting Cancer With Bifunctional Peptides: Mechanism of Cell Entry and Inciting Cell Death
Source: Cancer Sci. 2025 Mar 26;116(6):1730–44. doi: 10.1111/cas.70065 (PMC12127091; doi:10.1111/cas.70065)

**Supplementary figure S1:**

**Determining the escort protein candidates of the peptides** by a. verifying the inhibition of the genes by quantifying the mRNA of the respective genes, b. measuring the transcript of MYL12A and MYL12B in Caki-2 and SK-BR-3 by subtracting the Cq of the respective genes by the Cq of housekeeping gene. The viability of the knockdowns in b. Caki-2 and c. SK-BR-3 was measured after 1-2 h of peptides treatment the presence or absence of CPZ. d. The genes showed to affect the peptide toxic activity were knockdown concurrently in different arrangements and in the presence or absence of CPZ. The viability of the cells was measured after 1-2 h of peptide treatment. Data of a are displayed as means of the 2-dCq value ± SEM. Data of b is displayed as means of ∆Cq ± SEM. Both a-b used GAPDH as the housekeeping gene. Data of c-e are presented as means of relative viability ± SEM calculated from normalizing the treated samples to the untreated cell line of the corresponding wild type and knockdown (*n* = 3-9). *p < 0.05, **p < 0.01, ***p < 0.001 difference of b-d was calculated using Bonferroni‘s test between peptide treated samples and untreated samples. *p < 0.05, **p < 0.01, ***p < 0.001 determined the differences between knockdown and its particular CPZ treated group. *p < 0.05, **p < 0.01, ***p < 0.001 difference of e was calculated using Bonferroni‘s test between the simultaneous gene knockdown and their specific single gene knockdown group.


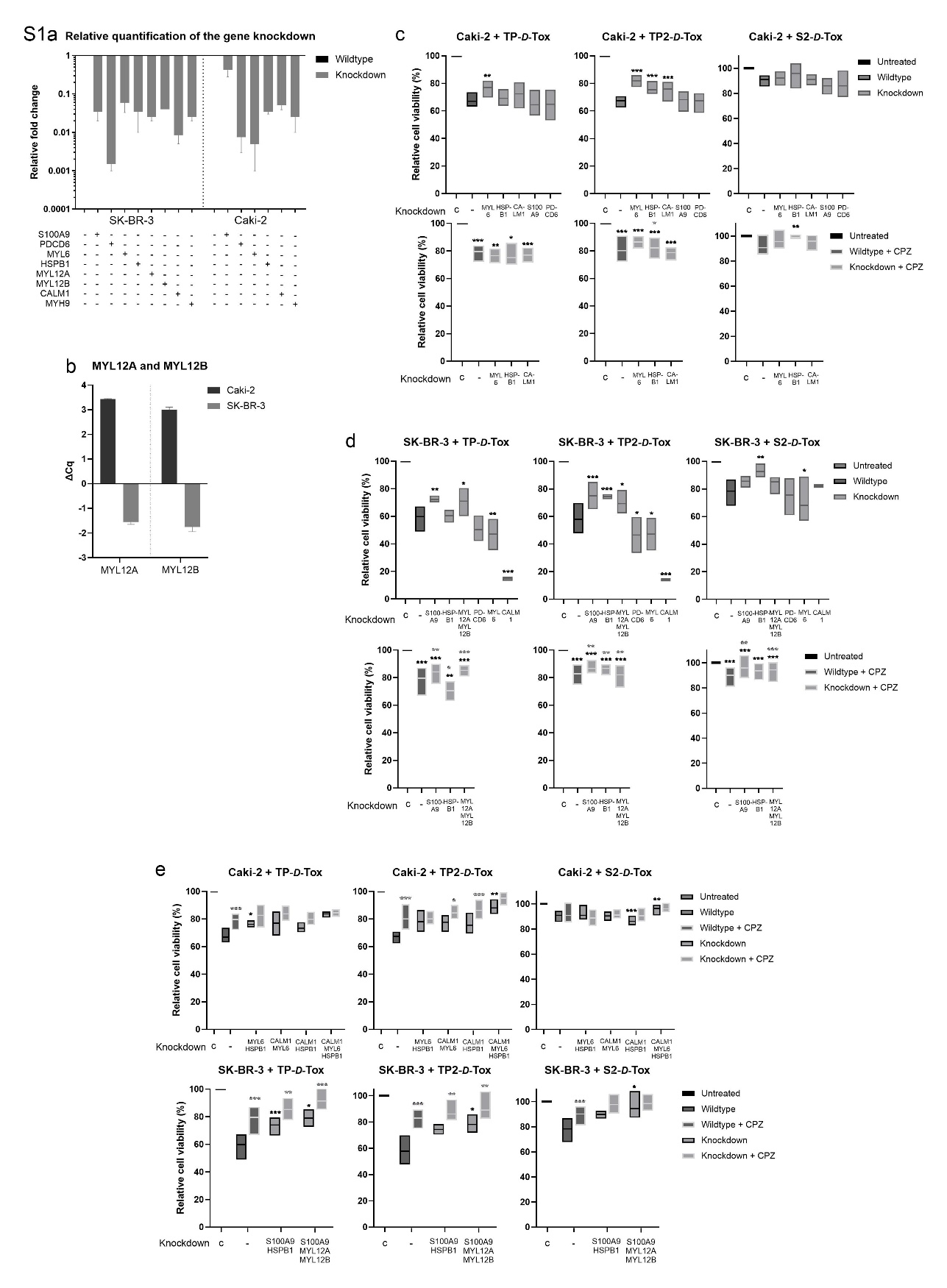

Supplement: Supplementary file 1 — Figure S1. Determining the escort protein candidates of the peptides. [file CAS-116-1730-s003.docx]
