# Supplementary material for: Targeting Cancer With Bifunctional Peptides: Mechanism of Cell Entry and Inciting Cell Death
Source: Cancer Sci. 2025 Mar 26;116(6):1730–44. doi: 10.1111/cas.70065 (PMC12127091; doi:10.1111/cas.70065)

**Supplementary figure S2:**

**Assessing the mPT following the peptide treatment** in a. Caki-2 and b. SK-BR-3 using MitoTrackers by staining the mitochondria of the cells. Red and blue population represented population measured after 3 and 22 h after treatment, respectively.


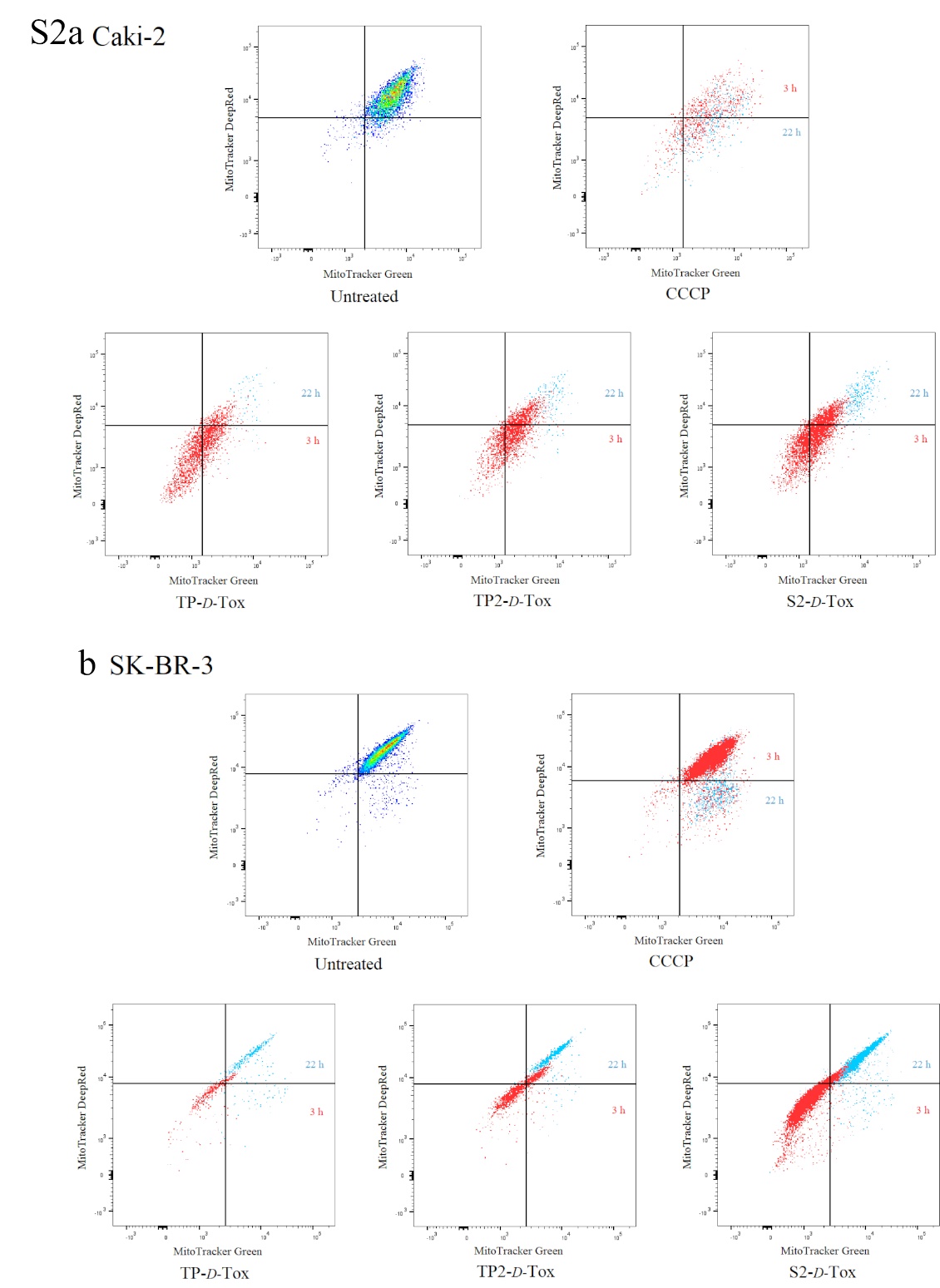

Supplement: Supplementary file 2 — Figure S2. Assessing mitochondrial membrane potential following the peptide treatment. [file CAS-116-1730-s001.docx]
