# Supplementary material for: Targeting Cancer With Bifunctional Peptides: Mechanism of Cell Entry and Inciting Cell Death
Source: Cancer Sci. 2025 Mar 26;116(6):1730–44. doi: 10.1111/cas.70065 (PMC12127091; doi:10.1111/cas.70065)

**Supplementary figure S3:**

**Measuring caspase 3 and 7 activity in the peptide-treated cells**. The caspases activity was detected using DEVD peptide in a. Caki-2 and b. SK-BR-3.


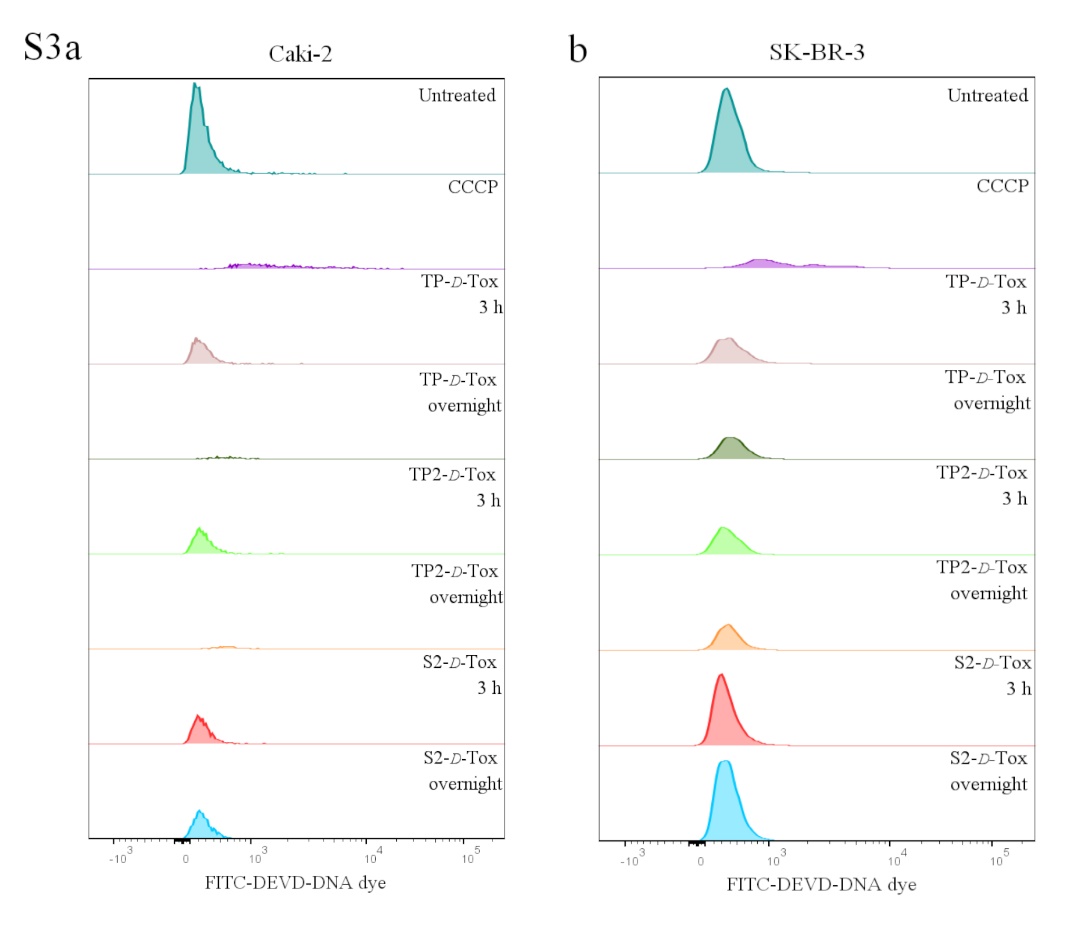

Supplement: Supplementary file 3 — Figure S3. Measuring caspase 3 and 7 activity in the peptide‐treated cells. [file CAS-116-1730-s006.docx]
