# Supplementary material for: Targeting Cancer With Bifunctional Peptides: Mechanism of Cell Entry and Inciting Cell Death
Source: Cancer Sci. 2025 Mar 26;116(6):1730–44. doi: 10.1111/cas.70065 (PMC12127091; doi:10.1111/cas.70065)

**Supplementary figure S4:**

**Single channel figures from the immunostaining of** a. colocalization analysis of MCF-7 and b. SK-BR-3, c. measuring MLKL oligomers in the plasma membrane of Caki-2 and d. SK-BR-3 Scale bar represents 50 μm.


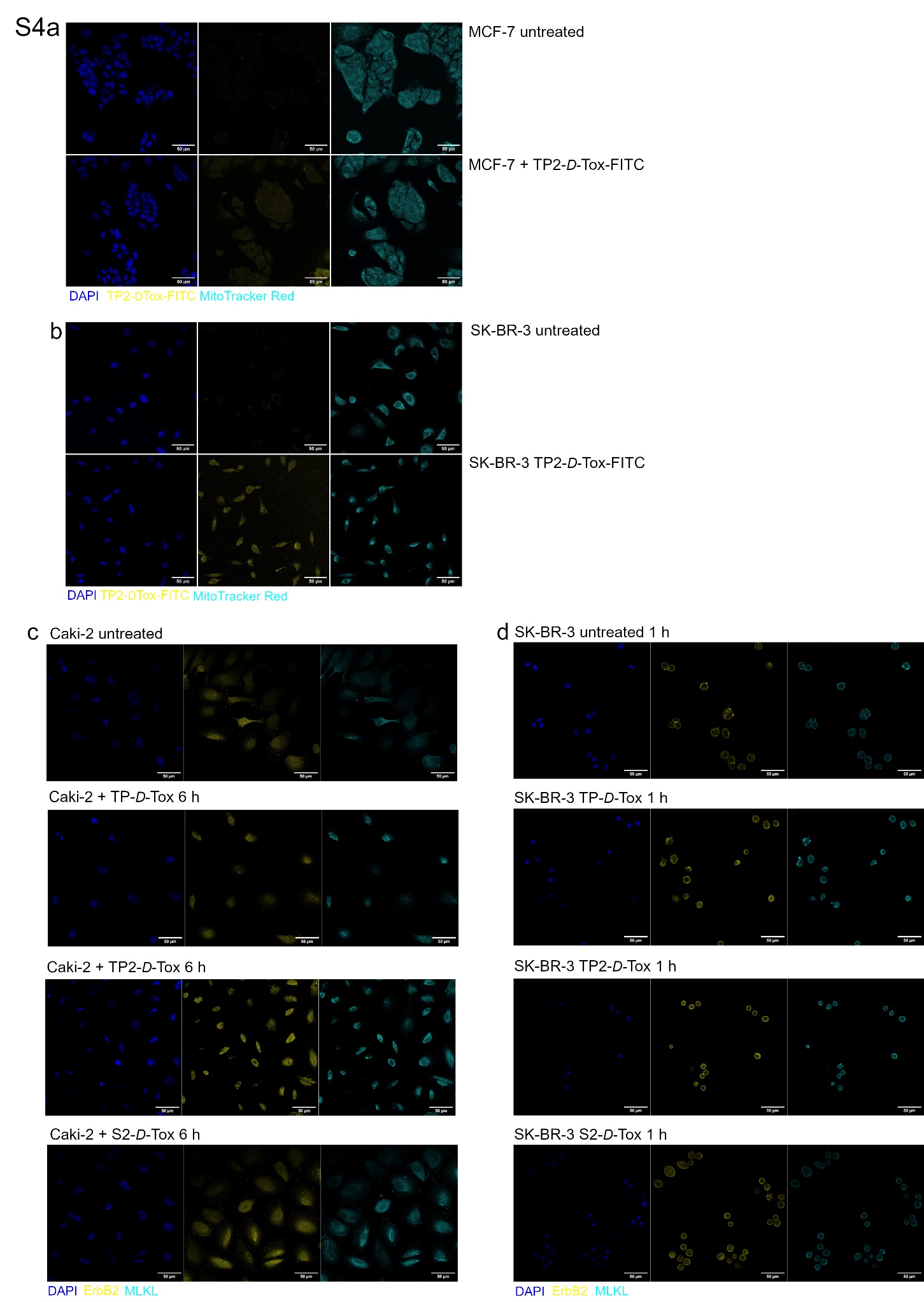

Supplement: Supplementary file 4 — Figure S4. Single channel figures from the immunostaining. [file CAS-116-1730-s005.docx]
