# Supplementary material for: Targeting Cancer With Bifunctional Peptides: Mechanism of Cell Entry and Inciting Cell Death
Source: Cancer Sci. 2025 Mar 26;116(6):1730–44. doi: 10.1111/cas.70065 (PMC12127091; doi:10.1111/cas.70065)
Supplement: Supplementary file 5 — Table S1. Information about tested cell lines. [file CAS-116-1730-s002.docx]

**Supplementary table S1:**

**Information about tested cell lines**

The cell lines used in this study were purchased from the American Type Culture Collection (ATCC) (Manassas, VA, USA) and tested for mycoplasma before used. All culture medium was supplemented with 10% heat inactivated FCS and 1% Penicillin-Streptomycin (Thermo Fisher Scientific). All cell lines were cultured at 37°C with 5% CO_2_ humidified atmosphere. The supplemented FCS was heat-inactivated before used at 56°C for 30 mins. All cancer cell lines to be used in the further experiments were maintained in culture within 10 passages. The CCD-18Co fibroblast was maintained and used in further experiments before reaching population-doubling time of 42.

| Cell lines | Medium |
| --- | --- |
| Breast adenocarcinoma  SK-BR-3  MDA-MB-231  MCF-7 | RPMI1640 (PAN Biotech, Aidenbach, Germany)  RPMI1640  MEM (PAN Biotech) |
| Renal carcinoma  A-498  Caki-2  A-704 | RPMI1640  RPMI1640  MEM |
| Melanoma  MDA-MB-435S | RPMI1640 |
| Neuroblastoma  Wac-02  Tet21N | RPMI1640  RPMI1640 |
| Ovarian carcinoma  A2780 | RPMI1640 |
| Prostate carcinoma  DU-145 | MEM |
| Colon fibroblast  CCD-18Co | MEM |
