# Supplementary material for: Targeting Cancer With Bifunctional Peptides: Mechanism of Cell Entry and Inciting Cell Death
Source: Cancer Sci. 2025 Mar 26;116(6):1730–44. doi: 10.1111/cas.70065 (PMC12127091; doi:10.1111/cas.70065)
Supplement: Supplementary file 6 — Table S2. List of DsiRNA utilized in the study. [file CAS-116-1730-s004.docx]

**Supplementary table S2:**

**List of DsiRNA utilized in the study**

The following DsiRNA were purchased from Integrated DNA Technologies (Corralville, IA, USA)

Table 2. List of DsiRNA employed for gene knockdown

| Gene | DsiRNA |
| --- | --- |
| S100A9 | hs.Ri.S100A9.13.1 (Integrated DNA Technologies, Coralville, IA, USA)  hs.Ri.S100A9.13.2 |
| PDCD6 | hs.Ri.PDCD6.13.8 |
| MYL6 | hs.Ri.MYL6.13.1 |
| HSPB1 | hs.Ri.HSPB1.13.1 |
| MYL12A | hs.Ri.MYL12A.13.1 |
| MYL12B | hs.Ri.MYL12B.13.2 |
| CALM1 | hs.Ri.CALM1.13.2 |
| MYH9 | hs.Ri.MYH9.13.1  hs.Ri.MYH9.13.2 |
